# Effect of Crystallization Modes in TIPS-pentacene/Insulating Polymer Blends on the Gas Sensing Properties of Organic Field-Effect Transistors

**DOI:** 10.1038/s41598-018-36652-1

**Published:** 2019-01-10

**Authors:** Jung Hun Lee, Yena Seo, Yeong Don Park, John E. Anthony, Do Hun Kwak, Jung Ah Lim, Sunglim Ko, Ho Won Jang, Kilwon Cho, Wi Hyoung Lee

**Affiliations:** 1 Department of Materials Science and Engineering, Research Institute for Advanced Materials, Seoul National University, Seoul, 08826 Republic of Korea; 2 Department of Organic and Nano System Engineering, Konkuk University, Seoul, 05029 Republic of Korea; 3 Department of Energy and Chemical Engineering, Incheon National University, Incheon, 22012 Republic of Korea; 4 Center for Applied Energy Research, University of Kentucky, Lexington, 40511 USA; 5 Center for Optoelectronic Materials and Devices, Korea Institute of Science and Technology, 02792 Seoul, Republic of Korea; 6 Department of Mechanical Design and Production Engineering, Konkuk University, Seoul, 05029 Republic of Korea; 7 Department of Chemical Engineering, Pohang University of Science and Technology (POSTECH), Pohang, 37673 Republic of Korea

## Abstract

Blending organic semiconductors with insulating polymers has been known to be an effective way to overcome the disadvantages of single-component organic semiconductors for high-performance organic field-effect transistors (OFETs). We show that when a solution processable organic semiconductor (6,13-bis(triisopropylsilylethynyl)pentacene, TIPS-pentacene) is blended with an insulating polymer (PS), morphological and structural characteristics of the blend films could be significantly influenced by the processing conditions like the spin coating time. Although vertical phase-separated structures (TIPS-pentacene-top/PS-bottom) were formed on the substrate regardless of the spin coating time, the spin time governed the growth mode of the TIPS-pentacene molecules that phase-separated and crystallized on the insulating polymer. Excess residual solvent in samples spun for a short duration induces a convective flow in the drying droplet, thereby leading to one-dimensional (1D) growth mode of TIPS-pentacene crystals. In contrast, after an appropriate spin-coating time, an optimum amount of the residual solvent in the film led to two-dimensional (2D) growth mode of TIPS-pentacene crystals. The 2D spherulites of TIPS-pentacene are extremely advantageous for improving the field-effect mobility of FETs compared to needle-like 1D structures, because of the high surface coverage of crystals with a unique continuous film structure. In addition, the porous structure observed in the 2D crystalline film allows gas molecules to easily penetrate into the channel region, thereby improving the gas sensing properties.

## Introduction

Organic field-effect transistors (OFETs) based on solution-processable conjugated small molecules or polymers are widely investigated owing to their popular application as components in electronic devices such as flexible displays, sensors, radio frequency identification tags, and logic circuits^1–5^. However, solution processing of small molecule semiconductors often poses certain problems: their strong π-π interactions induce non-uniform morphologies and dewetting of their films from the substrate, resulting in poor electrical performance of the devices^6,7^. In attempts to overcome these problems, the small-molecular semiconductors were blended with insulating polymers that have excellent film-forming properties and OFETs based on the films of such blends have been fabricated using various approaches^7–9^. Blending small-molecular semiconductors with insulating polymers effectively improves their processability, facilitates environmental stability of OFETs designed based on them, and provides blend films with no loss in the inherent charge carrier mobility due to the small molecule semiconductors through vertical phase-separation^9–11^. However, in order to improve the electrical performances of devices based on blend films, the phase-separation and crystallization of the small molecule/insulating polymer blends should be carefully controlled. Since the charge carriers in the active layer usually travel in a direction parallel to the dielectric surface, the formation of a vertical phase separation is ideal.

Recently, many researchers have studied the effects of the thermodynamic and kinetic factors that influence the vertical phase separation of organic small molecule/insulating polymer blends, i.e. the interaction energy with the substrate surface, Gibbs-free energy of the system, and the solidification kinetics, by blending small-molecule semiconductors with various types of insulating polymers. The vertical phase separation in small molecule semiconductor/insulating polymer is affected both by molecular and processing parameters; for example, the type and molecular weight (M_w_) of the insulating polymer, the surface tension of each component and substrate, and the spinning speed and time^9,12–16^.

Meanwhile, the effect of the spin-coating time on the OFET performance has not been studied extensively. In a majority of articles reporting investigations on solution-processed OFETs, the spinning time was varied from 3 to 120 s, depending on the volatility of the solvent used^10,17^. Field-effect mobilities of semi-crystalline polymer semiconductors could be improved by controlling the spin-casting time because the molecular orientation and π-π stacking interaction of the conjugated molecules could be enhanced by controlling the crystallization speed resulting from the residual solvent after spin-coating. For example, Na *et al*. examined the effects of residual solvent in spin-cast polythiophene film on the electrical properties of OFETs. They discovered that an optimum amount of residual solvent after spin-coating for a few seconds enhances the molecular order of the polythiophene film, resulting in high field-effect mobility. In small-molecule semiconductor/insulating polymer blends, controlling the residual solvent in spin-cast blend films is also important for inducing vertical phase-separation, while achieving control over the crystallization of small molecule semiconductor for high-performance OFETs^10,16,18^.

The electrical properties of OFETs such as the mobility, on-current, and threshold voltage are affected by the presence of gas molecules, biomolecules, and chemical analytes near the interface between the semiconductor and gate-dielectric^19–26^. Here, the analytes act as dopants to assist the charge transport or as traps to inhibit charge migration. In particular, gas molecules affect the charge transport due to their dipolar character. Because gas molecules should permeate the semiconducting layer to reach the interface between the semiconductor and gate-dielectric, the microstructure of a semiconducting layer is important with respect to the sensing properties of OFET gas sensors. For example, porous organic semiconductor films are recommended as active layers because the porous microstructure allows the analytes to reach the active layer more efficiently and reduce the time for the adsorption/desorption process with the active layer of the device^27,28^. Therefore, it is disadvantageous when the organic semiconductor film is thick and uniform from the viewpoint of a gas sensor. In this work, 6,13-bis(triisopropylsilylethynyl)pentacene (TIPS-pentacene) was used as an organic semiconductor owing to its high field-effect mobility and solution-processability. Moreover, polystyrene (PS) without any polar groups was employed as the insulating polymer. We investigated phase-separation and structural development of TIPS-pentacene/PS blends by changing the spin-coating time of the blend solution. The crystallization mode of TIPS-pentacene in TIPS-pentacene/PS blend was examined by considering the amount of residual solvent in the TIPS-pentacene/PS blend film after spin-casting. The electrical characteristics and gas sensing properties of the FET devices were correlated with the microstructure of the blend films, which is governed by two different crystallization modes.

## Results

### Optical observation of TIPS-pentacene/PS blend film formation

**Figure 1:**
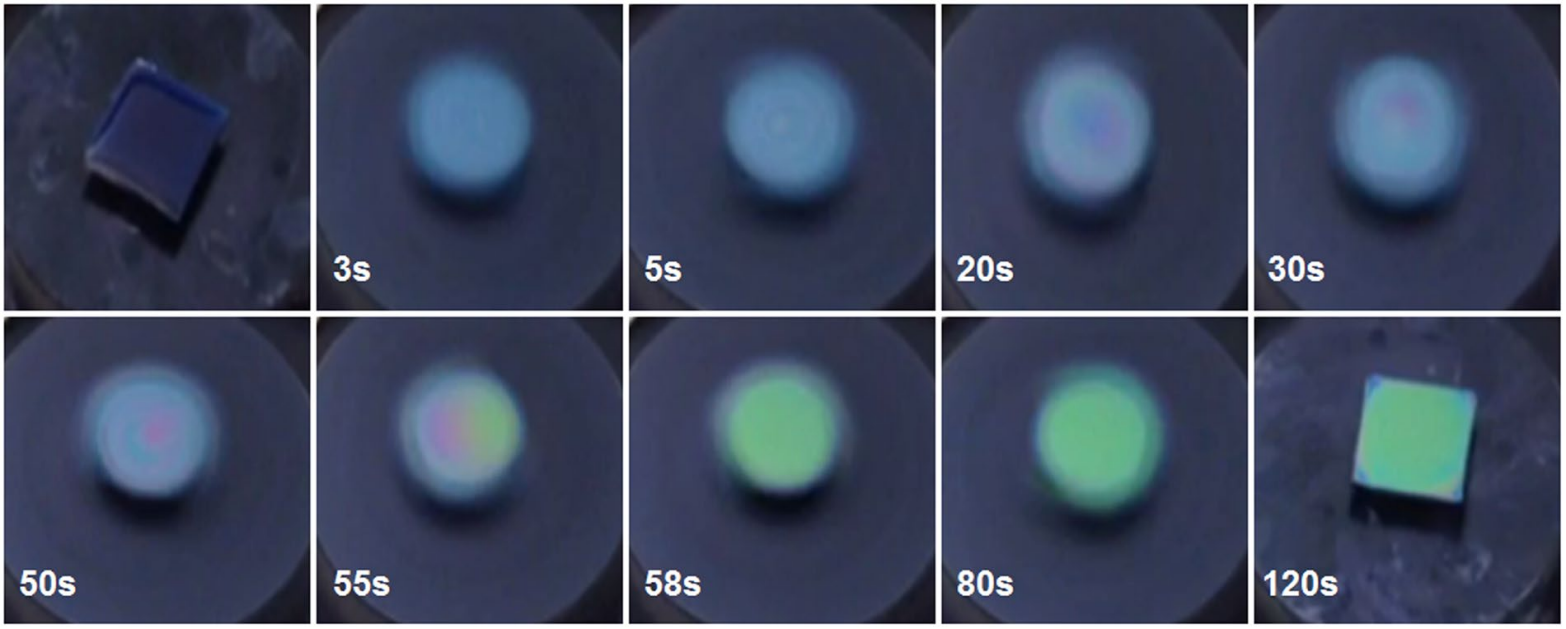
*In-situ* monitoring of the spin coating of a TIPS-pentacene/PS blend solution at specified times using optical microscopy. 20 mg/ml of the blend solution consisting of 10 mg of TIPS-pentacene and 10 mg of PS in 1,2-dichlorobenzene was used.

Figure 1 shows *in-situ* CCD camera images of the thin film formation process during the spin coating of a TIPS-pentacene/PS blend solution. Spin-coating is a dynamic process involving wetting, thinning, and solidification of the blend solution. Once the blend solution is casted on the substrate, the centrifugal force removes the excess solution and subsequent thinning of the transient wetting layer occurs by solvent evaporation. When the spinning of the substrate is stopped, the residual solvent in the spin-cast blend film begins to evaporate and phase-separation in the blend film and development of the microstructures of the TIPS-pentacene molecules are initiated due to the demixing of TIPS-pentacene and PS. The solvent used, high-boiling 1,2-dichlorobenzene which provides a low evaporation rate, is appropriate for maximizing the effects of the residual solvent. The color of the TIPS-pentacene/PS blend films began to change after a specific spin-casting time (critical spin-coating time of 50 s). After 58 s, the color of the blend films did not change significantly because of the fixed film thickness. Note that the color of the film reflects the thickness of the film due to optical contrast. Thus, it can be speculated that the amount of residual solvent does not decrease significantly after the critical spin-coating time. Because the amount of residual solvent is very small after the critical spin-coating time, thinning of the blend film is not facilitated by the ensuing spin coating process. PS is expected to phase-separate and lie at the bottom while TIPS-pentacene segregates at the air-film interface. The higher surface energy of the PS induces predominant interaction with SiO_2_, thereby resulting in its phase-separation near the SiO_2_ surface. In this study, we deliberately stopped the spin-coating at a given spin time and examined the effects of the residual solvent on the phase-separation and crystallization of TIPS-pentacene/insulating polymer blends, as discussed in the following section.

### Morphology of TIPS-pentacene/PS blend films

**Figure 2:**
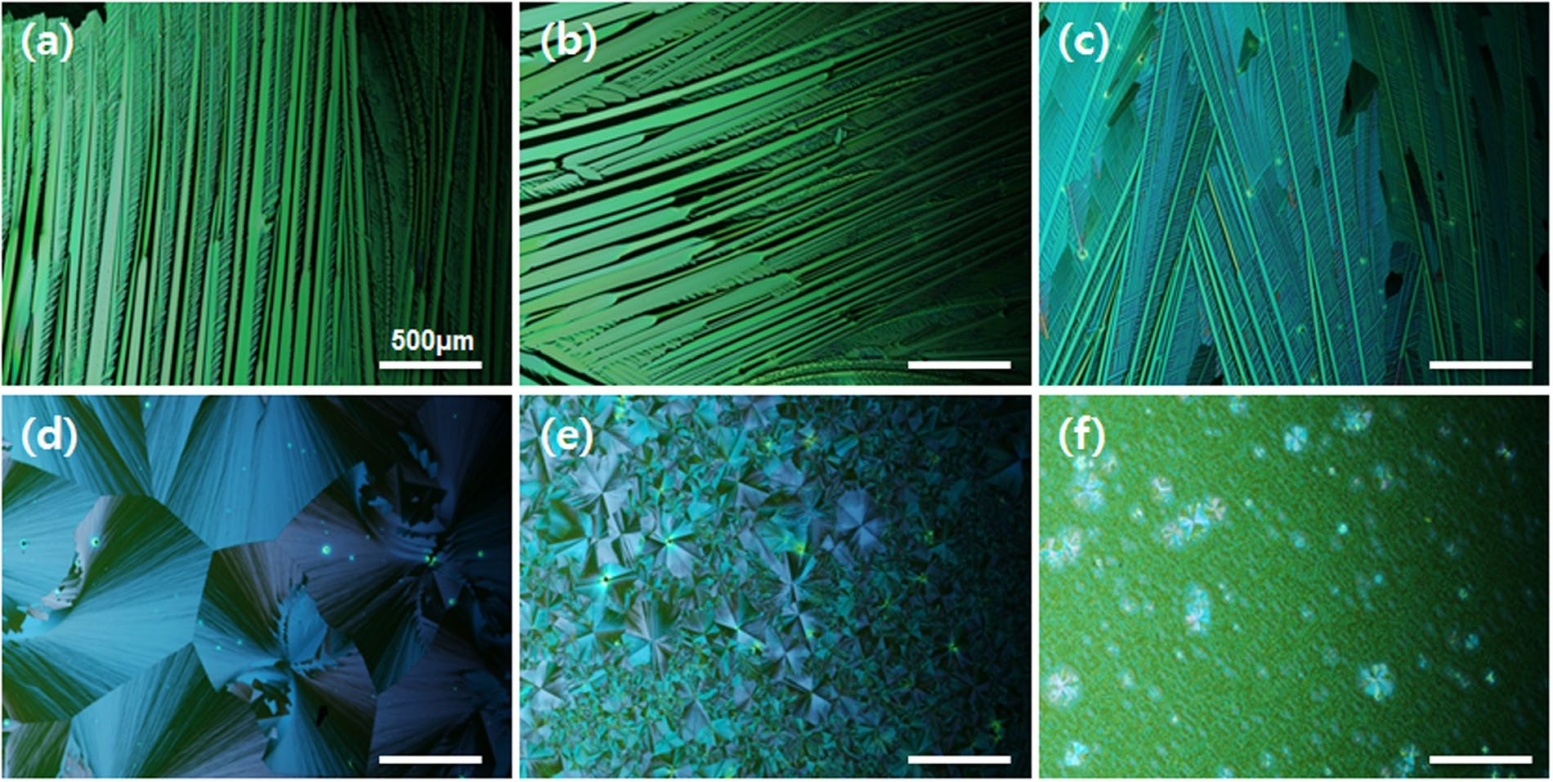
Polarized optical microscopy images of TIPS-pentacene/PS films spun-cast from blend solutions in 1,2-dichlorobenzene over different spin coating times: (**a**) 3 s, (**b**) 5 s, (**c**) 20 s, (**d**) 50 s, (**e**) 80 s, and (**f**) 120 s.

As depicted in Fig. 2, spin-coating times were changed from 3 to 120 s to examine the effects of the residual solvent on the morphological evolution of the TIPS-pentacene/insulating polymer blend. Polarized optical microscopy images of the TIPS-pentacene/PS blend films were recorded for different blend films obtained by varying the spin-coating time (3~120 s). The crystal shapes of the samples before and after a spin-coating time of 50 s are significantly different. When the spin coating time was shortened to 3 or 5 s, one dimensional (1D) TIPS-pentacene structures are clearly observed. The excess residual solvent over a short spin-coating time induces drying-mediated convective flow in a droplet, thereby leading to 1D growth of TIPS-pentacene crystals at the edge of the substrate. When the spin-coating time was increased to more than 50 s, crystal growth mode changed and two dimensional (2D) structures and spherulites with nucleation centers and grain boundaries are formed. The sample obtained with a spin-coating time of 50 s shows the largest 2D crystal grain size and a plate-like surface morphology compared to other samples. In addition, the grain size of the spherulites decreased with increasing spin time. An increase in the spin time to 80 s led to an increase in the grain boundary density, mainly due to the rapid crystallization of TIPS-pentacene facilitated by the small amount of residual solvent after a long spin-coating time. A further increase in the spin time to 120 s led to spherulite crystals with very small grains.

**Figure 3:**
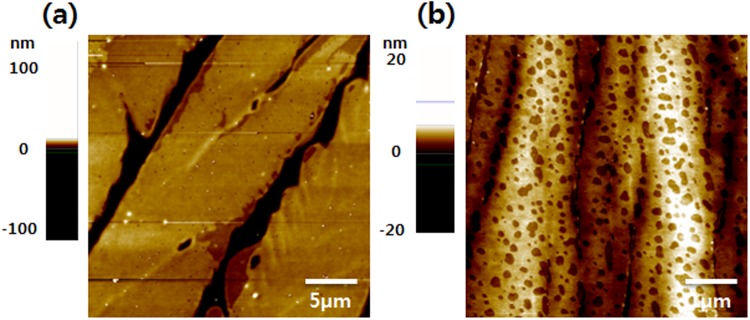
Atomic force microscopy (AFM) height images of TIPS-pentacene/PS blend films spun-cast for (**a**) 5 s, and (**b**) 50 s.

As depicted in Fig. 3, TIPS-pentacene/PS blend films were characterized with AFM to examine surface morphologies of the TIPS-pentacene crystals. AFM image of a sample obtained with a spin time of 5 s shows oriented 1D crystals with sharp edges (Fig. 3a). The thicknesses of the 1D crystals were measured to be in the range of 470–910 nm and there are gaps of width 1–3 μm between adjacent crystals (see Fig. S1 in Supporting information). Thus, strong π -π interaction between the TIPS-pentacene molecules leads to thick 1D crystals with empty spaces when the amount residual solvent in the blend film is high^29^. In contrast, the surface image of 2D crystals has a completely different morphology (Fig. 3b). The measured thickness of the 2D crystals is in the range of ~88 to 120 nm and a continuous film with high density of voids is observed. AFM images of both the films (spun-coat for 5 and 50 s) indicate that TIPS-pentacene is situated at the top of the blend film. Fig. S2 in Supporting information shows the cross-polarized optical microscopy images of TIPS-pentacene/PS blend films before and after the etching of TIPS-pentacene with *n*-hexane. The images clearly show that TIPS-pentacene at the top has been totally removed and the PS layer at the bottom does not contain TIPS-pentacene. Thus, a structure with TIPS-pentacene on top and PS at the bottom was formed regardless of the growth mode (1D *versus* 2D) of TIPS-pentacene.

### Structural development of TIPS-pentacene in TIPS-pentacene/PS blend films

**Figure 4:**
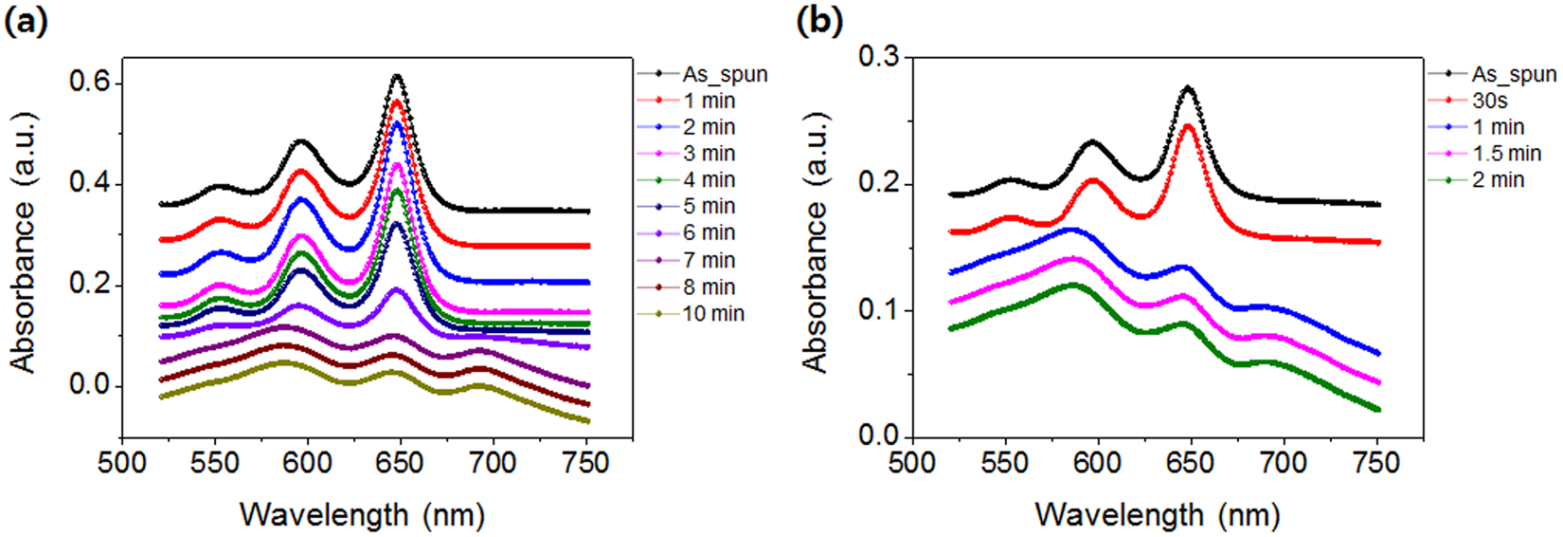
*In-situ* UV-Vis spectra of the TIPS-pentacene/PS blend films spun-cast for (**a**) 5 s and (**b**) 50 s.

To examine the crystallization kinetics of TIPS-pentacene molecules during solvent evaporation in spin-cast TIPS-pentacene/PS blend films, *in-situ* UV-Vis absorption spectra of the blend films obtained with different spin-coating times (5 and 50 s) were analyzed (Fig. 4). In the initial stage of solvent evaporation, absorption peaks centered at approximately 646, 593, and 550 nm were observed, which are due to the TIPS-pentacene molecules in the free state in solution^30^. When solvent evaporates from the blend films and the solution becomes viscous, TIPS-pentacene molecules self-assemble to form TIPS-pentacene crystals by spontaneous π-π interaction. At this stage, the absorption peaks red-shift to 692, 646, and 588 nm corresponding to the self-assembled TIPS-pentacene molecules in the solid state. The observed peak shifts are related to the enhanced packing of the TIPS-pentacene molecules^30^. As shown in Fig. 4a, in the blend films spin-coated for 5 s, the solution to solid transition occurs between 3 and 7 min. In contrast, in the blend films spin-coated for 50 s, the transition occurs between 30 s and 1 min. Because a blend film spun-coat for 5 s contains more residual solvent than that spun-coat for 50 s, the crystallization speed is slower for the former. However, the crystallization speed cannot solely account for the molecular orientation of TIPS-pentacene in the TIPS-pentacene/PS blend films. Note that the two different crystallization mechanisms govern the growth characteristics of TIPS-pentacene during solvent evaporation.

**Figure 5:**
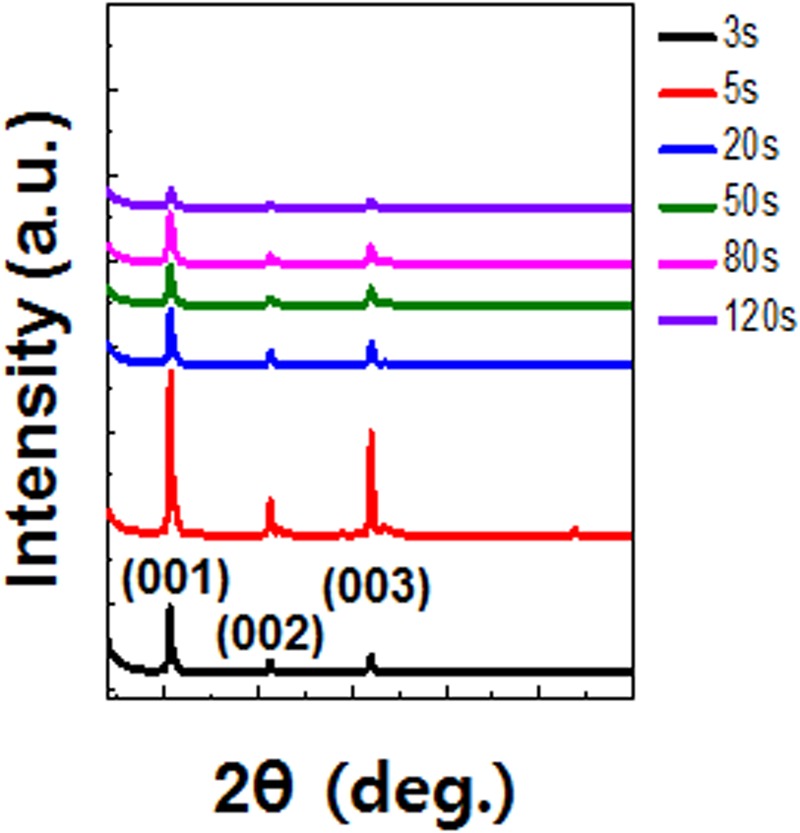
Normal-mode X-ray diffraction patterns of the TIPS-pentacene/PS blend films spun-cast for times ranging from 3 to 120 s.

To determine the crystalline structures of TIPS-pentacene in TIPS-pentacene/PS blends, normal-mode X-ray diffraction (XRD) patterns were obtained for the samples with different spin times (Fig. 5). All the TIPS-pentacene/PS films showed (0 0 l) diffraction peaks, implying that the TIPS-pentacene molecules were crystallized with TIPS groups aligned vertically on the substrate surface. This orientation is quite advantageous to increase the overlapping of neighboring pentacene molecules along the parallel direction. The peak intensity of the TIPS-pentacene/PS blend films gradually decreased with increasing spin-coating time. 1D crystals exhibit higher peak intensities for the out-of-plane direction compared to those of 2D crystals. Specifically, 1D crystals exhibit four times higher peak intensity compared to 2D crystals. This is directly related to an increase in the out-of-plane molecular layers in thick 1D crystals. Note that the thickness of a film with 1D crystals is more than six times higher than that with 2D crystals (Supporting information Fig. S1). Thus, the normal-mode XRD results indicate that 2D crystals are not as good as 1D crystals, in terms of crystalline ordering. We also measured two-dimensional grazing incidence X-ray diffraction patterns to confirm the crystal structures in more details (Supporting information Fig. S3). Sharp Bragg rods in 2D crystals indicated that in-plane orientation of 2D crystals is superior compared to the orientation of 1D crystals. Thus, it can be concluded that overall crystal perfectness of 2D crystals is higher than that of 1D crystals.

### Electrical properties of FETs based on TIPS-pentacene/PS blend films

In order to extract the charge carrier mobility from the transfer characteristics, capacitances of the dielectric layers were calculated by measuring thickness of the phase-separated PS layer at the bottom (Table 1). On one hand, the thickness of the PS layer is approximately 100 nm in the case of a blend film spun-coat for 3 and 5 s. On the other hand, the thicknesses of the blend films spun-coat between 20 and 120 s are 70 nm. Since the phase-separated PS and SiO_2_ (thickness of 300 nm) are serially connected, the total areal capacitance of the dielectric layer is calculated using the following equation:

$${C}_{PS}=k{\varepsilon }_{0}/t,1/{C}_{tot}=1/{C}_{PS}+1/{C}_{SiO2(10.8nFc{m}^{-2})}$$

Here, *k* is the dielectric constant of PS (2.6 was used in this work)^29^, *ε*_0_ is the permittivity in vacuum (8.85 × 10^−14^ F cm^−1^), and *t* is the thickness of the PS layer. The thicknesses of the PS layers and the calculated total areal capacitances according to the spin-coating time are summarized in Table 1.

**Table 1:** Thickness and areal capacitance of the PS layers in etched TIPS-pentacene/PS blend films that were spun-cast over different durations ranging from 3 to 120 s

Spin time [s]	t_Dielectric_ [nm]	C_tot_ [nF cm^−2^]
3	100	7.35
5	100	7.35
20	70	8.12
50	70	8.12
80	70	8.12
120	70	8.12

**Figure 6:**
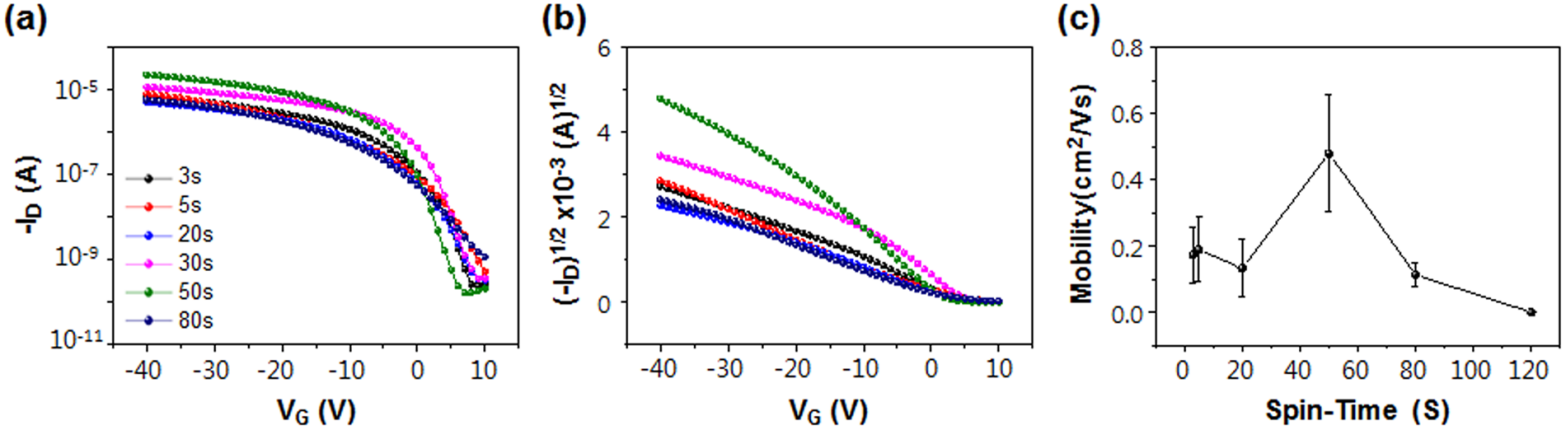
Electrical characteristics and a summary of the device performance. (**a**) −*I*_*D*_ and (**b**) (−*I*_*D*_)^*1/2*^ *versus V*_*G*_ for the FETs based on TIPS-pentacene/PS blend films spun-coat for different durations. *V*_*D*_ is fixed at −100 V. (**c**) Field-effect mobilities of the blend films as a function of the spin coating time.

To measure the electrical characteristics of the TIPS-pentacene/PS blend films, Au source and drain electrodes were deposited on the blend films and bottom-gate/top-contact FETs were fabricated. The transfer characteristics for a sweep voltage *V*_*G*_ ranging from 10 V to −100 V are shown in Fig. 6. Field-effect mobilities and current on/off ratios were extracted from the transfer characteristics and are shown in Table 2. The device based on TIPS-pentacene/PS blend film spun-coat during 50 s exhibits a maximum field-effect mobility of 0.653 cm^2^/V·s and average field-effect mobility of 0.480 cm^2^/V·s, which can be rationalized by analyzing the polarizing optical microscopic (POM) and AFM images (Figs 2 and 3). 2D spherulitic crystals with a large grain size of >1 mm are detected in the POM images of the blend films spun-coat for 50 s. In the film spun-coat over a longer time (80 s), the grain size of the 2D spherulites is decreased, resulting in a reduction in the field-effect mobility. In the POM and AFM images of the blend films spun-coat for 3 and 5 s, large-scale inter-crystal gaps between the 1D crystals lead to a lower field-effect mobility. Conversely, the POM and AFM images of films spun-coat for 50 s indicate that the crystals are not only large but also seamlessly connected. Thus, the charge carriers can efficiently travel from the source to drain, and accordingly, the field-effect mobility is nearly doubled. We surmise that the two different growth modes govern the crystallization behaviors of TIPS-pentacene, thereby leading to different electrical properties of FETs based on them.

**Table 2:** Electrical properties of FETs based on TIPS-pentacene/PS blend films spun-cast over durations ranging from 3 to 120 s

Spin time [s]	µ [cm^2^/(V·s)]		I_on_/I_off_
	average	max	
3	0.174	0.296	>10^5^
5	0.190	0.314	>10^5^
20	0.134	0.274	>10^5^
50	0.480	0.653	>10^6^
80	0.114	0.172	>10^5^
120	N/A	N/A	N/A

### Gas sensing properties of FETs based on TIPS-pentacene/PS blend films

**Figure 7:**
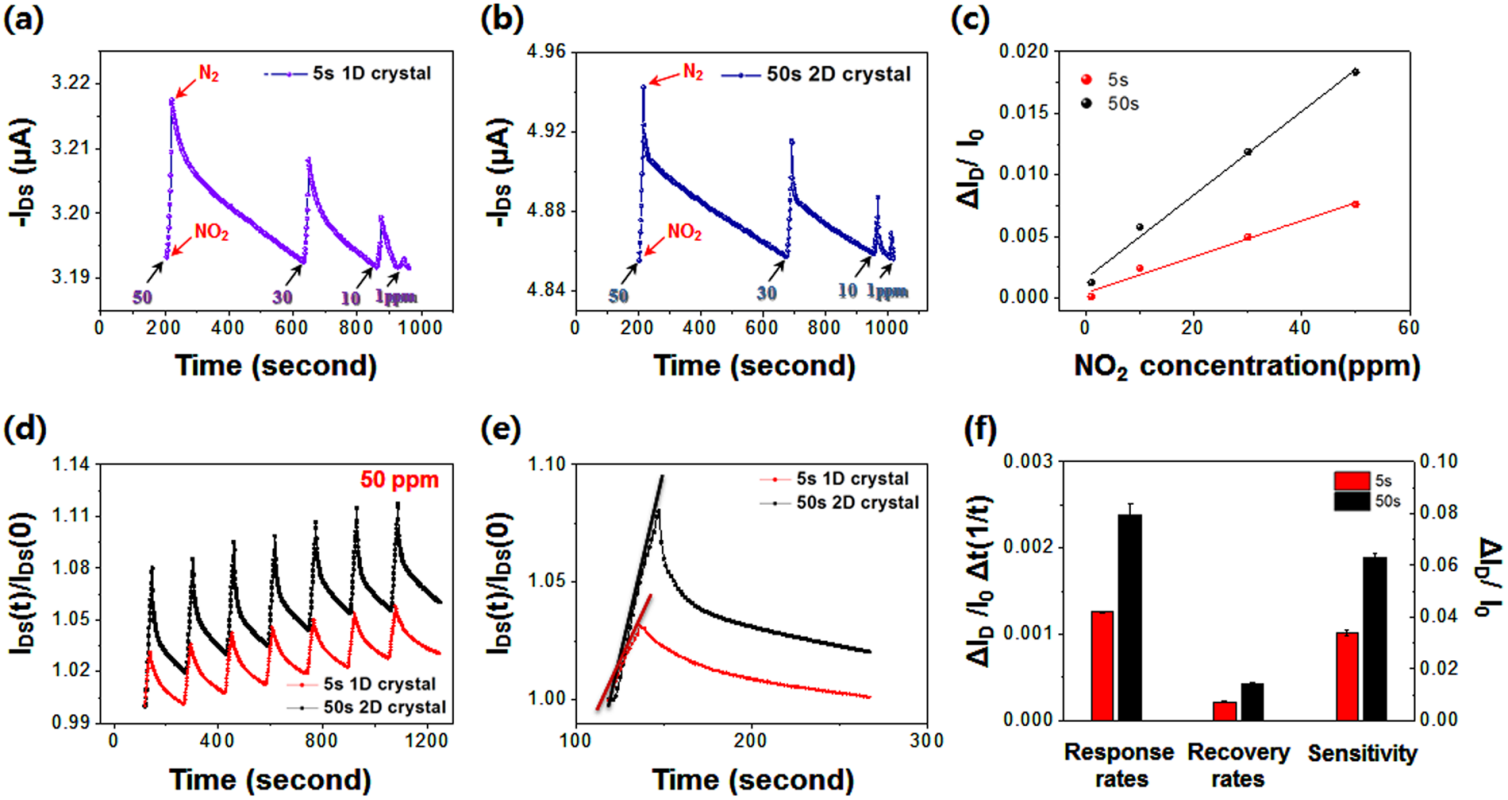
Gas sensing analyses of OFET-based gas sensors based on TIPS-pentacene/PS blend films. (**a,b**) *I*_*D*_ vs. time curve based on OFETs spun-cast for 5 and 50 s operated under N_2_ during the NO_2_ detection process. (**c**) Linear fit showing the relative response rate of the source-drain current (*ΔI*_*D*_*/I*_0_) as function of the NO_2_ concentration. (**d**) Repetitive sensing curves of OFET gas sensors based on the blend films upon exposure to successive pulses of NO_2_ (50 ppm) and N_2_. (**e**) Magnified analysis of (**d**). (**f**) Sensing parameters of TIPS-pentacene/PS sensors upon exposure to NO_2_ (50 ppm) and N_2_. All sensing experiments were carried out at *V*_*GS*_ = −10 V and *V*_*DS*_ = −10 V, respectively.

Figure 7 shows the gas sensing properties of FETs based on TIPS-pentacene/PS blend films at room temperature. The OFET gas sensors respond quickly under the exposure of NO_2_ gas, indicating the possibility of developing sensitive OFET gas sensors using TIPS-pentacene/PS blend films. The response mechanism can be explained by the dipolar character of NO_2_ with its strong electron withdrawing nature. When NO_2_ is situated at the interface between TIPS-pentacene and PS, extra hole carriers are accumulated by the electron withdrawing characteristics of NO_2_, thereby increasing the hole carrier density^31,32^. As shown in Fig. 7a,b, the dynamic change in the source-drain current was monitored using various cycles of NO_2_ /N_2_ exposures at different concentrations of NO_2_ (50, 30, 10, and 1 ppm). Excellent linear fits of the relative response rate of the source-drain current (*ΔI*_*D*_*/I*_0_) *versus* the concentration of NO_2_ indicate appropriate operation of the gas sensors (Fig. 7c). Figure 7d shows comparative response curves of TIPS-pentacene/PS gas sensors under the successive pulses of NO_2_ (50 ppm) and N_2_. Despite slight increases in the base currents, the repeatability of the response of both sensors over 7 cycles is excellent. Comparative plots in Fig. 7e show the difference in the sensing properties of the two gas sensors. Figure 7f summarizes the sensing performances of the TIPS-pentacene/PS gas sensors. The sensor based on 2D crystals performs better than that based on 1D crystals, in terms of the response rate, recovery rate, and sensitivity.

## Discussion

We have demonstrated that morphological and structural characteristics of films of blend of solution processable organic semiconductor, (6,13-bis(triisopropylsilylethynyl)pentacene (TIPS-pentacene), blended with an insulating polymer (PS) could be significantly influenced by the spin coating time. Although vertical phase-separated structures (TIPS-pentacene-top/ PS-bottom) were formed on the substrate regardless of the spin coating time, the spin time governed the growth mode of the TIPS-pentacene molecules which phase-separate and crystallize on the insulating polymer. Excess residual solvent in samples spun for a short duration induces a convective flow in the drying droplet, thereby leading to the growth of one-dimensional (1D) crystal of TIPS-pentacene, whereas for a prolonged spin-coating time, there is an optimum amount of the residual solvent leading to two-dimensional (2D) TIPS-pentacene crystals. We further demonstrate that the 2D spherulites of TIPS-pentacene are beneficial for improving the field-effect mobility of FETs compared to needle-like 1D structures, because of the high surface coverage of crystals with a unique continuous film structure. More importantly, a film with 2D crystals shows superior sensing properties compared to that containing 1D crystals.

**Figure 8:**
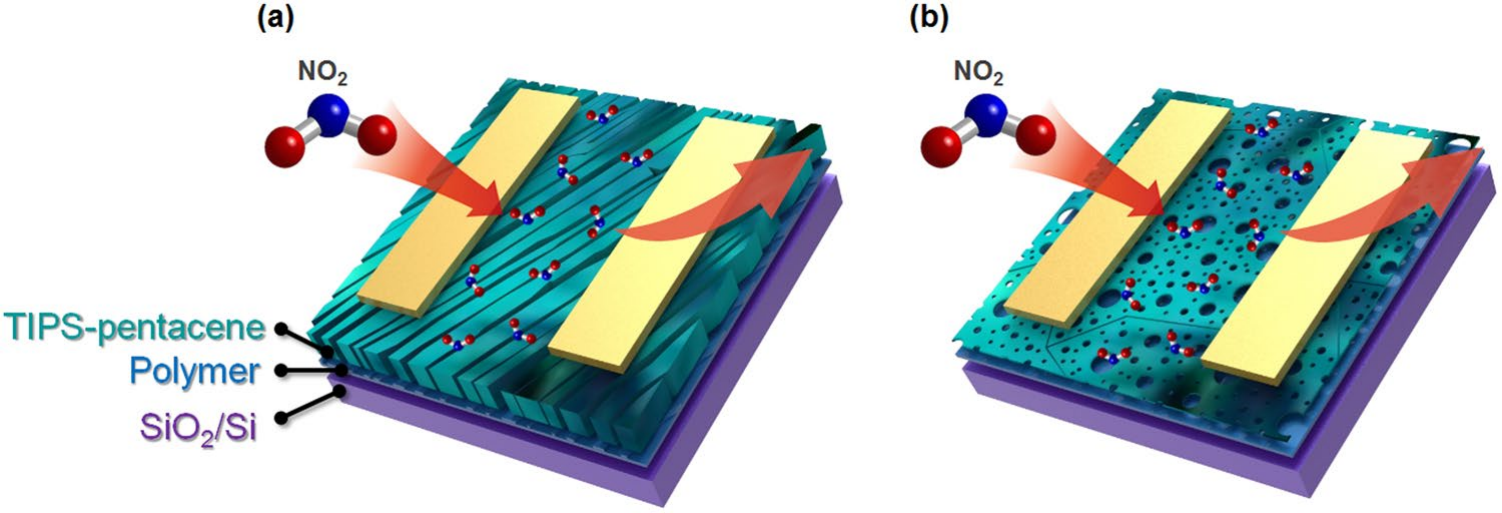
Schematic diagram showing gas sensing mechanism of TIPS-pentacene/PS FETs prepared with different spinning times: (**a**) 5 s, (**b**) 50 s.

Many previous studies have shown that the sensitivity of a sensor increases with decreasing thickness of the semiconducting layer. The thickness of TIPS-pentacene in the blend films is shown in Fig. S1 of Supporting information. In the case of the sample spun-coat for 5 s, the thickness of the TIPS-pentacene layer ranges from 500 nm to 1 μm, however, the sample spun-coat for 50 s has an average thickness of 110 nm. The relatively thin 2D crystals with a porous structure allowed the analyte gas molecules to easily penetrate the channel region, thereby leading to the high sensitivity under NO_2_ exposure (Fig. 8). In addition, the porous structure in 2D crystals allows the analyte gas molecules to escape from the channel region when a high concentration of N_2_ is introduced into the gas chamber. However, TIPS-pentacene films with thicknesses of the order of several hundred nanometers prohibit instantaneous recovery of the current. Achieving further control over the thickness of TIPS-pentacene by changing the concentration of the blend solution might enhance the sensing properties of the derived FET gas sensors. We noticed that highly sensitive NO_2_ gas sensors were recently reported by using ultrathin TIPS-pentacene film^32^.

## Conclusions

Spun-coat films of a TIPS-pentacene/insulating polymer blend were used as active layers in OFET gas sensors. The degree of phase-separation, crystallization of TIPS-pentacene, and field-effect characteristics of the films, as well as the gas sensing properties of the FETs derived from the films changed according to the spin-coating time. Vertical phase-separated structures with TIPS-pentacene structures on top and insulating polymer at the bottom formed spontaneously on the SiO_2_ substrates, owing to the difference in surface energy. Although vertical phase-separated structures formed on the substrate regardless of the spin-coating time, the spin time governed the growth mode of TIPS-pentacene molecules on the insulating polymer. The excess residual solvent in case of short spin-coating times such as 3 and 5 s induces convective flow in the drying droplet, thereby leading to 1D growth of TIPS-pentacene crystals. In contrast, an optimum amount of the residual solvent in films spun-coat for 50 s led to 2D growth of TIPS-pentacene crystals. Characterization of the surface microstructures revealed that the film with 1D crystals contains large-scale inter-crystal gaps whereas the film with 2D spherulites is continuous with a high density of voids. Because of the high amount of the residual solvent in films spun-coat over short durations (5 s), 1D crystals are obtained due to slow crystallization kinetics, in contrast with films obtained with longer spin-coating times that contain 2D crystals. The TIPS-pentacene spherulites with a large grain size are quite advantageous for increasing the field-effect mobility of FETs, and thus, the field-effect mobility and the on/off ratio were measured to be ~0.6 cm^2^/V·s and ~10^6^, respectively. FET gas sensors with 2D crystals exhibited better sensing properties than those with 1D crystals, mainly owing to the film thickness and a porous film structure. Thus, we conclude that varying the spin-coating time of organic semiconductor/insulating polymer blends affects the morphology, microstructure, and thickness of the film of the organic semiconductor as well as the phase-separation between the organic semiconductor and insulating polymer, and hence, optimizing the spin-coating time could be an effective way to improve the device characteristics of FETs and sensors.

## Materials and Methods

### Materials and sample preparation

TIPS-pentacene, PS (Mw = 230 kg mol^−1^) and 1,2-dichlorobenzene were purchased from Aldrich Chemical Co. Heavily doped silicon wafers containing a 300-nm-thick SiO_2_ layer purchased from Fine Science were used as substrates. The wafers were cleaned by sonication in acetone and isopropyl alcohol for 30 min each. Then, the wafers were rinsed quickly with isopropyl alcohol and dried with nitrogen gas, followed by 20 min UV ozone exposure. PS was dissolved in 1,2-dichlorobenzene at a concentration of 10 mg mL^−1^ and then TIPS-pentacene was dissolved in the PS solution to obtain a TIPS-pentacene/PS (1:1 w/w) blend solution (20 mg mL^−1^). The blend solution was spin-cast onto the silicon substrate at approximately 1000 rpm for different spin times. After spin coating, the samples were immediately placed in separate Petri dishes and wrapped with aluminum foil to prevent light illumination and to induce slow evaporation of the solvent in ambient air. For the top-contact OFET devices, Au source-drain electrodes (channel length: 150 μm and width: 1500 μm$){\rm{w}}$ere thermally evaporated through a shadow mask. After the deposition of source and drain electrodes, each device was electrically isolated by a mechanical scratch. For gas sensor measurements, top-contact FETs were fabricated by depositing Au source-drain electrodes (channel length: 100 μm and width: 2000 μm) to improve the efficiency of the wire packaging of the gas sensor. In order to selectively remove the TIPS-pentacene layer, the TIPS-pentacene/PS blend films were etched with *n*-hexane.

### Characterization

The morphologies of the films were characterized using an optical microscope (Nikon), scanning electron microscope (Helios NanoLab 660), and atomic force microscope (Park Scientific Instrument, Autoprobe-PC). UV-Vis absorption spectra were recorded using a UV-Vis spectroscope (Agilent Technologies, CARY 60). To measure the thicknesses of the TIPS-pentacene and PS layers, a surface profiler (AlphaStep AS-IQ) was used. The inner structure of the TIPS-pentacene/polymers blend films was characterized by normal mode X-ray diffraction (Rigaku, SmartLab). Current-voltage characteristics of all the FET devices were measured using a probe station and a Keithley 4200-SCS. In order to investigate the transfer curves the gate voltage was swept from *V*_*G*_ = 10 V to *V*_*G*_ = −100 V, while the source-drain voltage was maintained at *V*_*D*_ = −100 V. Gas sensing properties of OFET gas sensors were measured using a gas chamber (Precision Sensor System Inc., GASENTEST) at the fixed biases, *V*_*GS*_ = −10 V and *V*_*DS*_ = −10 V.

## Electronic supplementary material

Supporting Information
