# Supplementary material for: Effect of Crystallization Modes in TIPS-pentacene/Insulating Polymer Blends on the Gas Sensing Properties of Organic Field-Effect Transistors
Source: Sci Rep. 2019 Jan 10;9:21. doi: 10.1038/s41598-018-36652-1 (PMC6328639; doi:10.1038/s41598-018-36652-1)
Supplement: Supplementary file 1 — Supporting Information [file 41598_2018_36652_MOESM1_ESM.doc]

Supporting Information

Effect of Crystallization Modes in TIPS-pentacene/Insulating Polymer Blends on the Gas Sensing Properties of Organic Field-Effect Transistors

Jung Hun Lee^1, 2^, Yena Seo^2^, Yeong Don Park^3^, John E. Anthony^4^, Do Hun Kwak^2^, Jung Ah Lim^5^, Sunglim Ko^6^, Ho Won Jang^1^, Kilwon Cho^7^, and Wi Hyoung Lee^2*^

^1^ Department of Materials Science and Engineering, Research Institute for Advanced Materials, Seoul National University, Seoul 08826, Republic of Korea

^2^ Department of Organic and Nano System Engineering, Konkuk University, Seoul 05029, Republic of Korea

^3^ Department of Energy and Chemical Engineering, Incheon National University, Incheon 22012, Republic of Korea

^4^ Center for Applied Energy Research, University of Kentucky, Lexington 40511, USA

^5^ Center for Optoelectronic Materials and Devices, Korea Institute of Science and Technology, 02792, Seoul, Republic of Korea

^6^ Department of Mechanical Design and Production Engineering, Konkuk University, Seoul 05029, Republic of Korea

^7^ Department of Chemical Engineering, Pohang University of Science and Technology (POSTECH), Pohang 37673, Republic of Korea

* Correspondence and requests for materials should be addressed to W.H.L (email: [whlee78@konkuk.ac.kr](mailto:whlee78@konkuk.ac.kr))


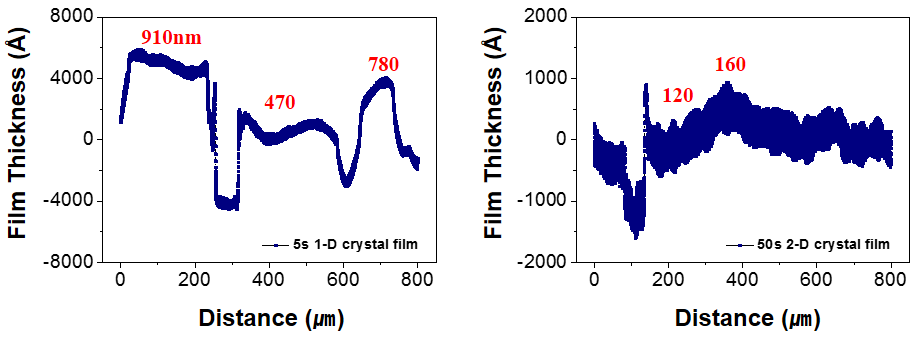


|  | 5 s | 50 s |
| --- | --- | --- |
| Film thickness | 737 (±137) nm | 111 (±21) nm |

**Figure S1.** Surface profiles of the TIPS-pentacene/PS blend films (5 and 50 s). The average film thickness of TIPS-pentacene was estimated by measuring the thickness on 10 arbitrary areas of each sample.


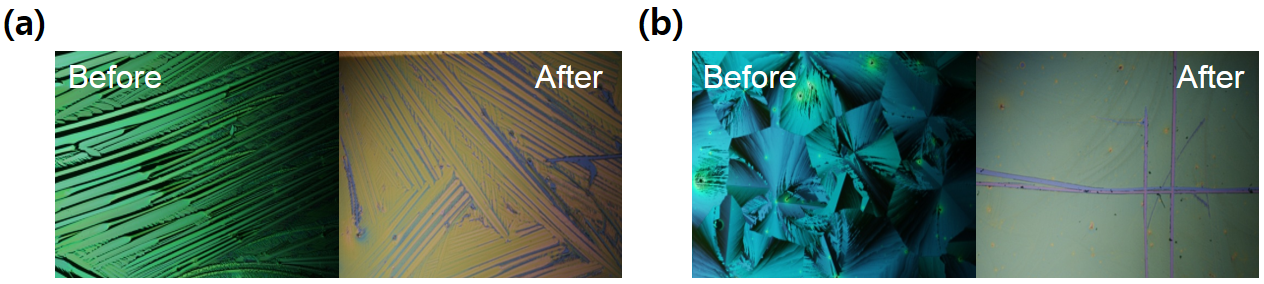


**Figure S2.** Cross-polarized optical microscopy images of TIPS-pentacene/PS blend films spun-coat for (a) 5 s and (b) 50 s before and after etching with n-hexane.


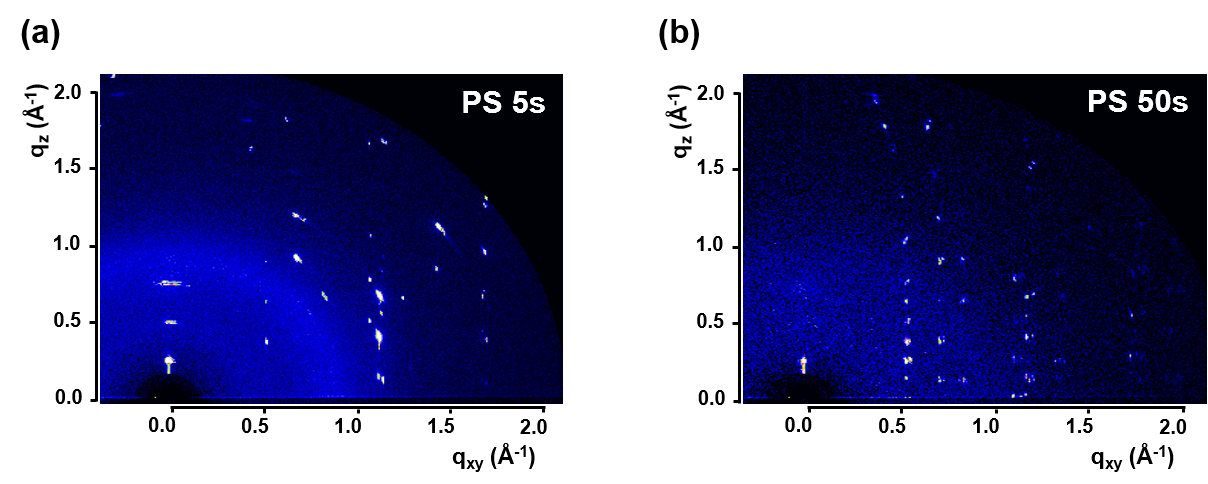


**Figure S3.** Two-dimensional grazing incidence X-ray diffraction patterns of TIPS-pentacene/PS blend films spin-cast for different spin coating times: (a) 5 s, (b) 50 s.


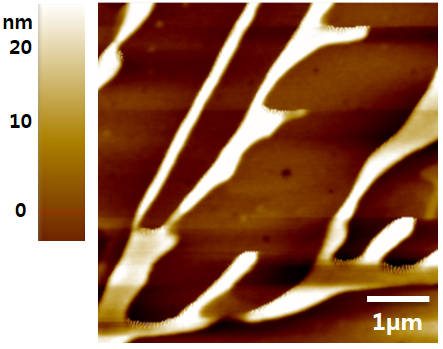


**Figure S4.** Atomic force microscopy (AFM) height image of a TIPS-pentacene/PS blend film (spin coating time of 50 s) after etching with n-hexane.


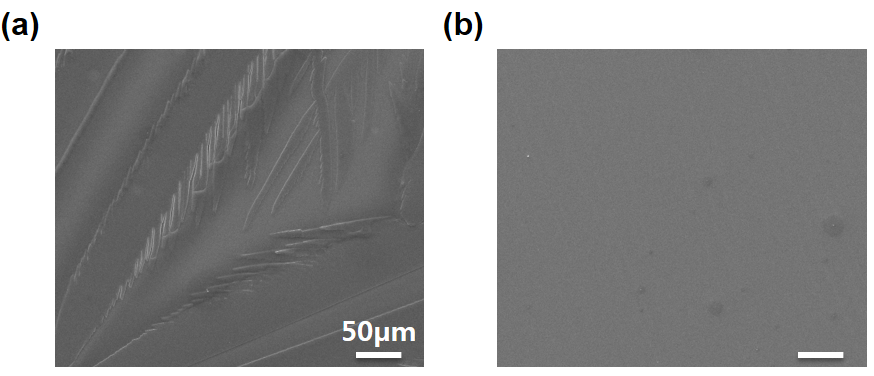


**Figure S5.** Field-emission scanning electron microscopy (FESEM) images of TIPS-pentacene/PS blend films spun-cast over a duration of (a) 5 s and (b) 50 s.
